# Amygdala‐predominant α‐synuclein pathology accelerates CA1 neuron loss in Alzheimer’s disease

**DOI:** 10.1002/alz.086519

**Published:** 2025-01-03

**Authors:** Klara Gawor, Sandra O. Tomé, Simona Ospitalieri, Alicja Ronisz, Rik Vandenberghe, Markus Otto, Christine von Arnim, Philip Van Damme, Mathieu Vandenbulcke, Matthew Blaschko, Dietmar Rudolf Thal

**Affiliations:** ^1^ Laboratory for Neuropathology, KU Leuven, Leuven Belgium; ^2^ Leuven Brain Institute, Leuven Belgium; ^3^ Department of Neurosciences, KU Leuven, Leuven Belgium; ^4^ UZ Leuven, Leuven Belgium; ^5^ Laboratory for Cognitive Neurology, KU Leuven, Leuven Belgium; ^6^ University Hospital Ulm, Ulm Germany; ^7^ University Medical Center Göttingen, Göttingen Germany; ^8^ Neuropsychiatry unit, KU Leuven, Leuven Belgium; ^9^ Center for Processing Speech & Images, KU Leuven, Leuven Belgium

## Abstract

**Background:**

In 43‐63% of symptomatic Alzheimer’s disease (AD) patients, there is an observed accumulation of misfolded alpha‐synuclein (αSyn). Two primary αSyn subtypes have been identified based on the underlying spreading pattern of this pathology: caudo‐rostral and amygdala‐predominant. Interactions between pathological TDP‐43, Tau, and αSyn can aggravate their spread and aggregation. The amygdala might serve as an incubation hub due to its early involvement in these pathologies. Here, we tested our hypothesis that the amygdala‐predominant αSyn pathology contributes to limbic degeneration, including the hippocampus, and increases the burden of other protein aggregates.

**Methods:**

We conducted a neuropathological analysis of 291 autopsy brains from both demented and non‐demented elderly individuals. Neuronal density in the CA1 region of the hippocampus was quantified from hematoxylin‐stained slides. We semi‐quantitatively evaluated αSyn‐pathology severity in six brain regions, stratifying cases into two spreading patterns. In 99 AD cases, we also assessed limbic‐predominant age‐related TDP‐43 encephalopathy neuropathological changes (LATE‐NC) stages and the proportion of pTau affected neurons in CA1.

**Results:**

We identified an association between amygdala‐predominant αSyn, but not caudo‐rostral, with decreased neuronal density in CA1 (P‐value<0.001). AD patients with amygdala‐predominant patterns showed higher LATE‐NC stages than those without αSyn (adj. P‐value = 0.015), while those with caudo‐rostral patterns had fewer neuritic plaques (adj. P‐value = 0.023) and pTau than pure AD (adj. P‐value = 0.038). Using structural equation modeling, we found that the relationship between αSyn and CA1 cell loss is indirect, mediated through hippocampal pTau and TDP‐43.

**Conclusions:**

Our findings indicate that the amygdala‐specific αSyn pattern, but not the rostro‐caudal one, may indirectly contribute to hippocampal neurodegeneration by enhancing the aggregation and toxicity of TDP‐43 and pTau. Furthermore, our study reveals differences in neuropathological characteristics between AD patients with two spreading patterns. These findings underscore the significance of identifying co‐morbid pathologies in AD patients and the necessity for further research on distinct αSyn subtypes.

